# Experimental Study on the Temperature-Dependent Static, Dynamic, and Post-Dynamic Mechanical Characteristics of Municipal Solid Waste

**DOI:** 10.3390/ma17164012

**Published:** 2024-08-12

**Authors:** Zejin Wang, Shuyu Hu, Jiaxin Zhou, Peng Cui, Ying Jiang

**Affiliations:** 1 School of Economics and Management, Nanjing Tech University, Nanjing 211800, China; 18625087602@163.com; 2 Merchant Marine College, Shanghai Maritime University, Shanghai 200135, China; hushuyushishenxian@163.com (S.H.); 202230410002@stu.shmtu.edu.cn (J.Z.); 3 College of Ocean Science and Engineering, Shanghai Maritime University, Shanghai 200135, China; 4 Department of Engineering Management, School of Civil Engineering, Nanjing Forestry University, Nanjing 210037, China; cui@njfu.edu.cn; 5 School of Management Engineering, Jiangsu Urban and Rural Construction Vocational College, Changzhou 213147, China

**Keywords:** recycling municipal solid waste, static mechanical properties, dynamic mechanical properties, temperature

## Abstract

Municipal solid waste (MSW) has huge potential to be recycled as construction material, which would have significant benefits for environmental conservation. However, the cornerstone of this undertaking is a solid comprehension of the mechanical response of MSW in real-world engineering locations, taking into account the effects of stress levels and temperature. In this paper, well-mixed MSW samples were sieved and crushed to produce standardized specimens in cylindrical molds. A series of static, dynamic, and post-cyclic shear tests were conducted on the MSW at temperatures ranging from 5 °C to 80 °C with normal stresses of 50 kPa, 100 kPa, and 150 kPa. The experimental findings demonstrate that the static, dynamic, and post-cyclic mechanical response of MSW presents temperature range-dependency; temperature variation between 5 °C and 20 °C affects MSW’s mechanical reaction more than variation in temperature between 40 °C and 80 °C under various stress settings; at 5 °C~80 °C, the static peak shear strength of MSW is the highest, being followed by the post-cyclic peak shear strength, while the dynamic peak shear strength is the lowest; the sensitivity of the dynamic shear strength of MSW to temperature variation is the largest, being followed by the post-cyclic peak shear strength, and the static peak shear strength is the lowest.

## 1. Introduction

During the last decades, with economic development and an increasing population, the produced amount of municipal solid waste (MSW) has risen significantly in urban and rural areas [1,2]. Landfills are the main way to deal with MSW, and are widely constructed around the world. However, the construction of landfills occupies huge land resources. Also, the existence of toxic materials in MSW causes serious pollution of the environments surrounding landfills [3,4,5,6]. Incineration, composting, pyrolysis, and other MSW treatment technologies will cause varying degrees of damage to the environment. Using MSW as construction materials is a promising solution to recycle MSW which can effectively solve the above challenge and avoid environmental pollution [7,8,9,10]. The stability and bearing capacity of the foundation of a building directly affect its safety and service life, and the quality and performance of the foundation materials have a crucial impact on the stability and bearing capacity of the foundation. The samples of MSW in previous studies have advantages of low compressibility, high strength, corrosion resistance, and low cost [8,9,10]. The prepared MSW samples can be recycled and used as building materials for foundations and road embankments. They can also be used in golf course surfaces to provide a base for vegetation placement. Also, it is convenient to obtain MSW and easy to prepare it into foundation materials. The mechanical response of the foundation is essential to ensure its long-term operation safety. Therefore, it is significant to study the mechanical properties of MSW samples.

In engineering sites, the external and internal environmental temperature is variable, and the MSW will inevitably experience temperature loadings [11,12,13,14]. Due to the organic components in MSW, such as rubber, textiles, plastic, etc., the exothermic reaction of waste biodegradation in MSW widely occurs, meaning that the inside temperature of MSW can reach 80 °C [15,16,17,18,19]. MSW contains many thermal softening materials, such as synthetic high-molecular polymer, tissues, etc. [20,21,22]. This results in the softening of MSW in elevated temperatures, which inevitably causes the obvious weakening of the MSW’s mechanical properties [23,24,25,26,27,28,29]. However, the existing research has mainly paid attention to the influence of its composition [30], the reinforced method [31], its buried duration [32], and its moisture content [33] on the mechanical properties of MSW, while investigation of the impact of temperature on the mechanical response of MSW is rare due to the relative rareness of temperature-controlled large mechanical measurement apparatus used on MSW [34,35,36,37,38,39,40,41]. Thus, a systematical study of the temperature-dependent mechanical properties of MSW should be conducted which can promote the recycling of MSW as construction materials.

In reality, engineering projects usually experience diverse stress conditions, such as monotonic shear, cyclic shear, those resulting from overlaying construction materials, earthquakes, vehicles, etc. [34,35,36,37,38,39]. Due to the different stress loading forms, the static and dynamic mechanical responses of MSW under monotonic and cyclic shear loading, respectively, have obvious differences [40,41,42]. In the same stress loading magnitude, the MSW under cyclic shear loading should be more likely to fail than that under monotonic shear loading [43,44,45]. Also, due to the viscoplasticity of MSW, its stress history has a non-ignorable influence on its mechanical behaviour [46,47,48,49,50,51,52,53,54]. This can result in the pre- and post-cyclic monotonic shear mechanical response of MSW being different to some extent after experiencing cyclic shear loadings [55,56,57,58,59,60,61]. The current study about the mechanical properties of MSW mainly focuses on the static mechanical characteristics of MSW subjected to monotonic shear loading [62,63,64,65,66,67]. For instance, Lapeña-Mañero et al. [68] conducted monotonic triaxial shear tests on mechanical and biological treatment MSW, and their research indicates that pre-treatment can obviously alter the static mechanical properties of MSW. Liu et al. [69] investigated the shear strength of calcium-containing waste and sludge mixtures by carrying out static direct shear tests, and pointed out that the shear strength of the mixtures reaches the mechanical requirement of landfill cover. However, relevant reports about the dynamic and post-cyclic shear mechanical properties of MSW are seldom, let alone comparison between the static, dynamic, and post-cyclic shear mechanical properties of MSW while considering temperature effects [40,41,70,71]. Consequently, it is imperative to undertake comprehensive research and comparative analysis of the temperature-dependent mechanical response of MSW under various stress conditions, encompassing monotonic shear, cyclic shear, and post-cyclic shear. This endeavor aims to gain a profound understanding of the material’s mechanical performance across a spectrum of loading scenarios and temperatures.

Based on the analysis above, temperature variations have a significant effect on the mechanical properties of construction materials applied to engineering [18,72,73,74]. However, considering the effect of temperature, there are few reports on the static, dynamic, and post-cycling shear mechanical properties of MSW [70,71]. This study reports on a series of static, dynamic, and post-cyclic shear experiments on MSW at 5 °C~80 °C. The experimental results are useful for analyzing and comparing the static, dynamic, and post-cycling shear mechanical responses of MSW at different temperatures, normal stresses, shear amplitudes, and numbers of cycles. The results of this research will be of considerable value in promoting the recycling of MSW as building materials, which will be of great benefit to environmental protection.

## 2. Experimental Program

### 2.1. Experimental Apparatus

In this research, temperature-controlled static, dynamic, and post-cyclic shear tests on MSW were conducted by adopting temperature-controlled shear equipment [4,5,6,72]. This equipment is designed on the basis of the existing large-scale shearing equipment, which is manufactured in Shanghai, China. The traditional shearer has no temperature control function; thus, we added a temperature control system. The instrument mainly consists of an external temperature chamber and an internal shear system. During the tests, experimental temperature can be controlled at a steady value in a temperature range from −50 °C to 200 °C during a long duration (7 days).

### 2.2. Test Materials

The adopted MSW sample was directly collected from Laogang Landfill, located in Shanghai City, which is the largest landfill in China. It deals with about 70% of the MSW in Shanghai. The composition of the MSW in different landfills is indeed different. The Laogang Landfill is located in Shanghai, thus the MSW collected comes from the city. In addition, the Laogang Landfill is the largest landfill in Asia. Therefore, the composition of the MSW samples collected from the landfill is representative, and the feasibility and applicability of using MSW as an urban building material can be well-investigated. In addition, the waste composition may vary over time, which could affect the results of this study. Therefore, the composition of MSW in this paper refers to the latest literature on the pretreatment method of MSW [75], and the sampling ratios used in actual projects. The collected fresh MSW was transferred to a sieve to separate the large-dimensional board, plastics, papers, etc. The screened MSW was crushed and the crushed MSW was mixed well to ensure the representativeness of the test sample. In accordance with the requirements of the shear test, the uniformly mixed MSW samples were made into standardized specimens using cylindrical molds. Among other parameters, the standardization of the samples included density control, moisture content control, particle grading parameter control, etc. The composition of the prepared MSW was 6.39% metal and glass, 19.63% plastic, 17.42% paper, wood, and fiber, 19.20% organic matter, and 37.36% waste residue. The unit weight of the MSW sample was 9.65 kN/m^3^. The composition of the prepared MSW included 17.42% paper, wood, and fiber, which was easily absorbent. The properties of these materials change when they absorb water, which can affect the mechanical properties of MSW [76]. Therefore, the level of moisture content is important and affects the mechanical properties of MSW. In this study, the moisture content of each MSW specimen was strictly controlled at 39.30% to represent the actual engineering conditions. As mentioned before, the moisture content of the MSW specimens was standardized prior to testing; thus, the collection of MSW during hot or humid periods had no effect on its mechanical properties.

The stability and bearing capacity of the foundation directly affect the safety and service life of the building, and the quality and performance of the foundation materials have a crucial impact on the stability and bearing capacity of the foundation. MSW samples have the advantages of low compressibility, high strength, corrosion resistance, good thermal insulation, a low cost, and high processability [8,9,31,32,33]. The prepared MSW samples can be recycled and used as building materials for foundations and other projects. Also, it is convenient to obtain MSW and easy to prepare it into foundation materials. The application of MSW in foundation materials is environmentally friendly and has great social and economic effects, which provides a reference for the sustainable development of MSW treatment. 

### 2.3. Experimental Procedure

The interior measurements of the designed apparatus’s above and bottom shear boxes are 300 mm in length, 300 mm in width, and 200 mm in height. The shearing area is 300 mm by 300 mm. In the tests, the bottom shear box was filled out by the selected MSW sample, firstly, in four equal incremental layers (25 mm thickness each). The MSW sample filling process was then carried out using the identical arrangement of the top and lower shear boxes. The MSW specimens in the shear boxes were prepared by using a treatment of 16 light compactions per layer before each set of experiments, based on the number of compaction times stated in the standards and the number of compaction times used in engineering applications [77]. By fixing the upper shear box and moving the lower one, the relative movement between the two separate positions of the shear box caused shearing of the MSW. 

When the installation of the MSW sample was completed, the cabin door of the external-environment temperature chamber was closed, and the inside temperature of the chamber was adjusted to a certain value. MSW is prone to deformation or decomposition at high temperatures and to be brittle or lose strength at low temperatures [78,79,80]. Based on the extant literature, the extreme temperature of the ground surface for actual construction projects is 80 °C in the East China region. In order to take this situation into account, this study used temperatures ranging from 5 °C to 80 °C. Then, normal stress was imposed on the MSW from the upper loading plate on the sample. Based on available studies and with reference to the maximum and minimum normal stresses that foundations may be subjected to in actual projects that utilize MSW, normal stresses of 50 kPa, 100 kPa, and 150 kPa were selected to cover the range of normal stresses that occur during the service period of actual projects. After the consolidation of the MSW sample under the normal stress for 3 h, the shearing on the MSW was initiated. In this research, different stress statuses of monotonic, cyclic, and post-cyclic undrained shearing were conducted, with a shearing displacement rate of 1 mm/minute. For the monotonic shearing, the MSW sample was sheared along a monotonic direction with a displacement magnitude of 55 mm. For the cyclic shearing, the MSW sample was subjected to 10 cycles of cyclic shear loading (3 mm displacement). For the post-cyclic shearing, initially, the MSW sample was subjected 10 times to cyclic shear loading, as mentioned previously, and then the sample was sheared along a monotonic direction (55 mm displacement). The specific test scheme is listed in Table 1.

In earlier experiments, the experimental results for the mechanics of MSW showed a low degree of dispersion, and it was shown that the data obtained from a single experimental measurement can accurately reflect the mechanical properties of MSW [78,79,80,81]. Therefore, the mechanical results in this article are the measurements from a single test. In addition, the authors referred to the standard and previous literature for the preparation and standardization of the MSW specimens. The method of sample preparation used in this test has been widely recognized, and thus had little effect on mechanical properties of the MSW. Compared to MSW from actual construction projects, the mechanical properties of both are similar; thus, the authors did not set up a control experiment [77,78,79].

## 3. Results and Analysis

### 3.1. The Temperature-Dependent Static Mechanical Performance of MSW under Monotonic Shear Loading

**Figure 1:**
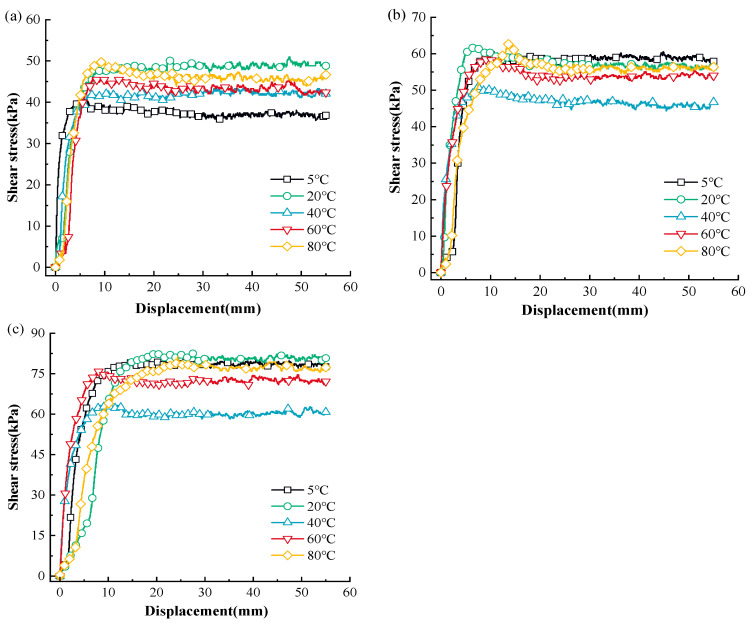
The displacement and shear stress relationship of MSW under monotonic shear loading: (**a**) 50 kPa; (**b**) 100 kPa; (**c**) 150 kPa.

**Figure 2:**
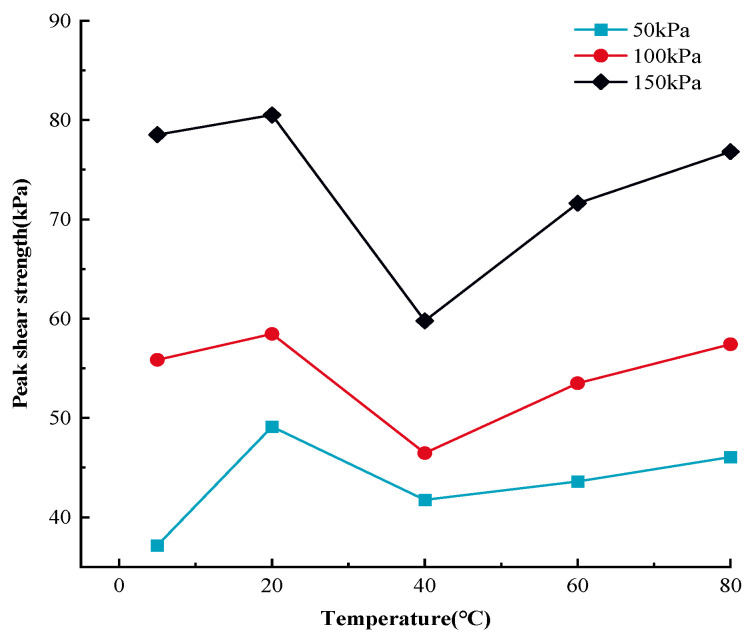
The correlation curves between MSW peak shear strength and temperature.

Figure 1 shows the displacement and shear stress relationship of MSW during monotonic shear loading at various temperatures, whereas Figure 2 shows the association curves between the temperature and the peak shear strength of MSW.

Based on Figure 1 and Figure 2, the MSW exhibited a displacement (strain) hardening behavior in all tests, without an obvious post-peak reduction in shear strength. This is in line with the regularity found in previous studies on the mechanical properties of MSW [81,82]. The displacement and shear stress relationship of the MSW, shows a temperature dependency at all temperatures studied. Additionally, the MSW mechanical response variation rules under various normal stresses are comparable and temperature range-dependent. More precisely, the peak shear strength rises between 5 °C and 20 °C and between 40 °C and 80 °C, but the peak shear strength falls between 20 °C and 40 °C. For instance, at 50 kPa normal stress, the MSW peak shear strength increases from 37.15 kPa to 49.12 kPa and from 41.75 kPa to 46.05 kPa at the temperature ranges from 5 °C to 20 °C and from 40 °C to 80 °C, respectively, while at 20 °C~40 °C, the MSW peak shear strength is reduced from 49.12 kPa to 41.75 kPa. Furthermore, compared to other temperature ranges, the MSW shear strength is more susceptible to temperature fluctuation at 20 °C~40 °C. For instance, at 150 kPa normal stress, the MSW shear strength is reduced by 25.77% from 80.52 kPa to 59.77 kPa when the temperature rises from approximately 20 °C to 40 °C, while the MSW shear strength ascends by 2.52% and 19.83% at the ranges from 5 °C to 20 °C and 40 °C to 60 °C, respectively. Moreover, it is noted that the MSW shear strength is more sensitive to temperature changes at high normal stress than it is at low normal stress. Taking the variation rules of the MSW shear strength at 40 °C~60 °C as an example, the shear strength rises by 19.83% from 59.77 kPa to 71.63 kPa, by 15.17% from 46.46 kPa to 53.51 kPa, and by 4.41% from 41.75 kPa to 43.59 kPa under 150 kPa, 100 kPa, and 50 kPa normal stress, respectively. Moreover, at different normal stresses, the MSW peak shear strengths all reach the maximum and minimum values at the temperatures of 20 °C and 40 °C, respectively. Regarding the temperature-dependent sensitivity of the MSW peak shear strength to normal stress variation, with a rise in temperature, the sensitivity firstly decreases and then continually rises, reaching the minimum value at a temperature of 40 °C.

### 3.2. The Temperature-Dependent Dynamic Mechanical Performance of MSW under Cyclic Shear Loading

**Figure 3:**
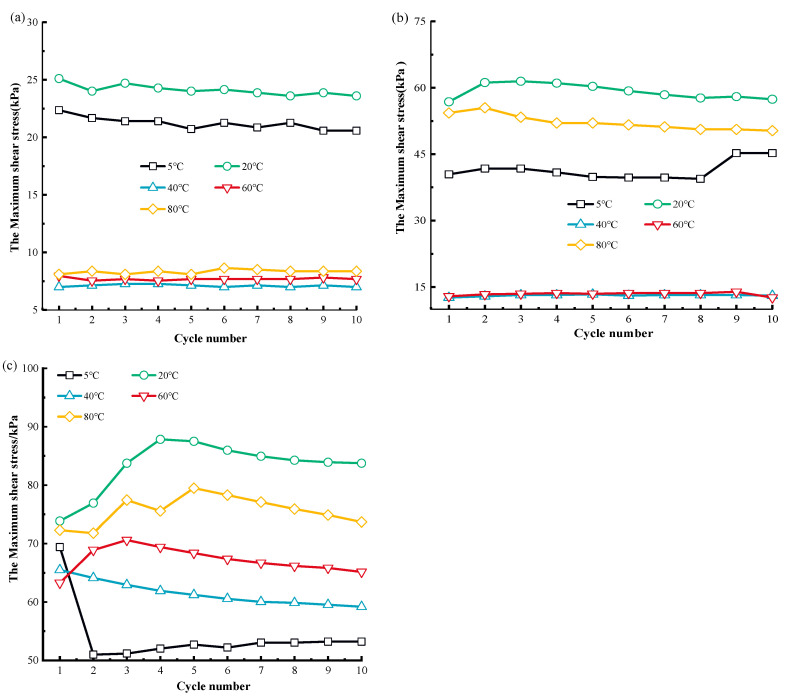
The correlation curve between the cycle shear number and the maximum shear stress: (**a**) 50 kPa; (**b**) 100 kPa; (**c**) 150 kPa.

Figure 3 shows the correlation curves of the maximum MSW shear stress (the maximum shear stress in a single cyclical shear loading) under cyclical shear loading at varying temperatures.

**Figure 4:**
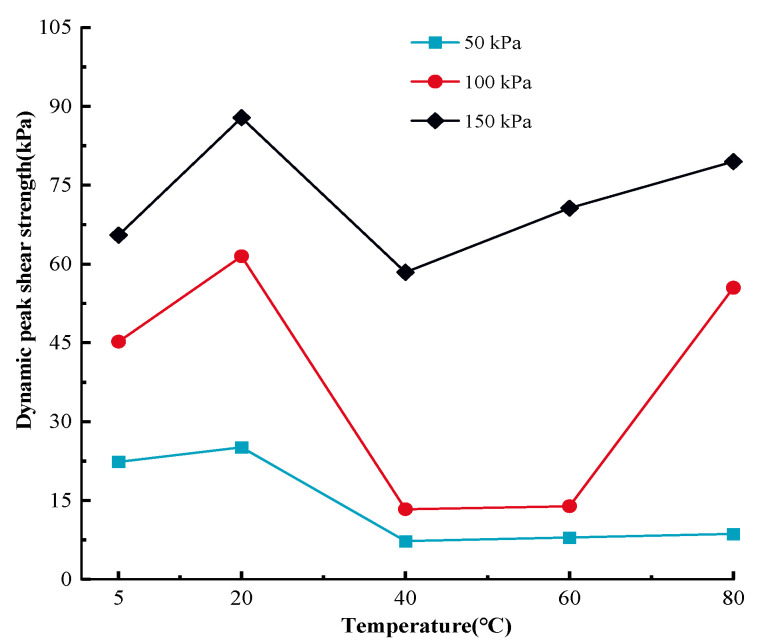
Dynamic peak shear strength versus temperature curves for MSW.

The dynamic MSW mechanical response exhibits a notable temperature sensitivity, as seen in Figure 3. There is a clear divergence between the maximum shear stresses of the MSW at various temperatures. When temperatures climb in the region of 5 °C~80 °C, the greatest MSW shear stress typically declines, with the exception of the maximum MSW shear stress at 40 °C. As an illustration, the ultimate MSW shear stress in the initial loop is 22.36 kPa, 25.11 kPa, 7.96 kPa, and 8.1 kPa at 50 kPa normal pressure for temperatures of 5 °C, 20 °C, 60 °C, and 80 °C respectively. Also, with the rise in normal pressure, the differences between the ultimate MSW shear stresses at different cyclic shearing numbers increases. The maximum shear stress of MSW at various cycle numbers is generally in the range of 50–100 kPa normal pressure, and the maximum shear stress decreases with increasing cycle times [83]. However, the correlation curves between the ultimate shear stress and cycle frequency at 150 kPa normal pressure showed significant fluctuations, and there was no clear pattern between the maximum shear stress and the number of cycles. Some studies in the literature have given similar results [84,85]. Figure 4 presents relationship curves related to the MSW peak shear strength to illustrate the temperature-sensitive mechanical response of MSW under dynamic loading.

Based on Figure 4, except for the dynamic peak shear strength at 20 °C~40 °C, the MSW dynamic shear strength gradually reduces with rises in temperature. For instance, at 150 kPa, the MSW shear strength rises from 65.49 kPa to 87.83 kPa in the 5 °C~20 °C temperature range, and from 58.41 kPa to 79.48 kPa in the 40 °C~80 °C temperature range. To be more precise, the dynamic peak shear strength is more susceptible to temperature changes in the 5 °C~20 °C region than to those in the 60 °C~80 °C range. It should also be noted that the temperature-dependent MSW peak shear strength reaches its maximum and minimum values at the temperatures of 40 °C and 80 °C, respectively. Previous studies have demonstrated that plastic waste creates potential slip surfaces within the waste body [86]. Thomas et al. [87] conducted large-scale shear strength tests (1000 mm × 1000 mm) by using natural MSW samples and showed that the shear strength decreases with increasing plastic content in the MSW. Therefore, it is speculated that the dynamic shear strength of MSW decreases with an increasing temperature, mainly because the temperature affects the mechanical properties of the plastic in MSW. The test apparatus used in this paper did not have a visual window, which would have enabled us to ensure a constant temperature environment in the environmental chamber, thus not allowing for a better observation of the deformation of the MSW samples as well as the changes in the shear zone. In the future, the authors will work on solving the limitations of the test apparatus.

When examining the dynamical mechanical response of a material, two essential factors to consider are its damping coefficient and dynamic shear strength [88,89,90,91]. The dynamic shear strength can reflect the ability of materials to withstand dynamic loading, which can be obtained according to Equation (1).

(1) $$K=\frac{K_{1}+K_{2}}{2}=\frac{\tau_{m1}+\tau_{m2}}{2\Delta_{a}}$$

where, *K*_1_ and *K*_2_ are the shear rigidity along the initial and opposite shear directions, respectively; *τ_m_*_1_ and *τ_m_*_2_ are the ultimate shear stress along the initial and opposite shear direction, accordingly; Δ*_a_* is the shear displacement magnitude.

The damping ratio presents the vibration damping capability, as shown in Equation (2). The closer the value of the damping ratio approaches 0, the smaller vibration damping of the material is [92,93,94,95]. The higher the damping ratio value, the stronger the vibration damping ability.

(2) $$D=\frac{D_{1}+D_{2}}{2}=\frac{1}{2}\left(\frac{A}{4\pi A_{1}}+\frac{A}{4\pi A_{2}}\right)=\frac{A}{4\pi \Delta_{a}}\left(\frac{1}{\tau_{m1}}+\frac{1}{\tau_{m2}}\right)$$

where, *D*_1_ and *D*_2_ are the damping ratio along the initial and opposite directions, respectively; *A* is the hysteresis circuit area; *A*_1_ and *A*_2_ are the triangular areas formed by the positive and negative peaks and the coordinates of the (0, 0) point in the hysteresis loop, respectively.

**Figure 5:**
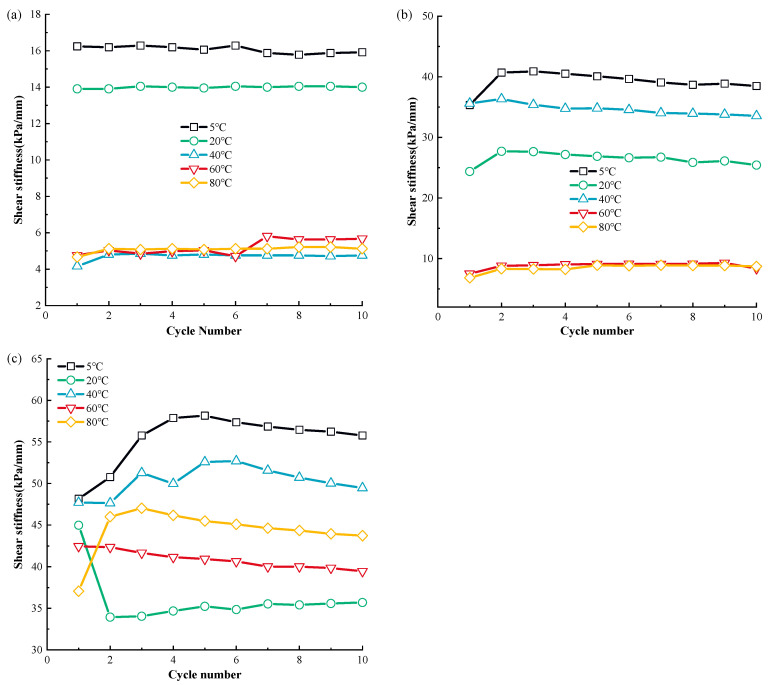
The relationship curve of MSW dynamic shear rigidity and cycle number under different normal stress conditions: (**a**) 50 kPa; (**b**) 100 kPa; (**c**) 150 kPa.

The correlation curves in Figure 5 show the link between the cyclic shear frequency and dynamic MSW shear strength at various temperatures.

The temperature significantly affects the dynamic MSW shear rigidity, as seen in Figure 5. The MSW shear stiffness generally decreases with an increasing temperature under various normal stress, with the exception of the shear rigidity in the temperature range of 20 °C–40 °C. For instance, at 100 kPa, the shear stiffness in the first cycle decreases from 35.60 kPa to 24.36 kPa when the temperature ascends from 5 °C to 20 °C, and the shear rigidity drops from 36.33 kPa to 6.81 kPa when the temperature is elevated from 40 °C to 80 °C. Additionally, the sensitivity of the MSW dynamic shear stiffness at 5 °C~20 °C is higher than that at 60 °C~80 °C. For instance, at 50 kPa normal stress, the MSW dynamic shear rigidity in the initial cycle decreases by 14.36% from 16.23 to 13.90 when the temperature ascends from 5 °C to approximately 20 °C, while the shear stiffness is reduced by 2.17% from 4.60 to 4.50 when the temperature elevates from 60 °C to 80 °C. Regarding the variation rules of MSW’s dynamic shear rigidity during cyclic shear frequency, it is found that, except for the first number, the dynamic MSW shear stiffness during the subsequent cycle frequency is similar. This indicates that the cyclical shear number has a marginal influence on the shear rigidity. 

**Figure 6:**
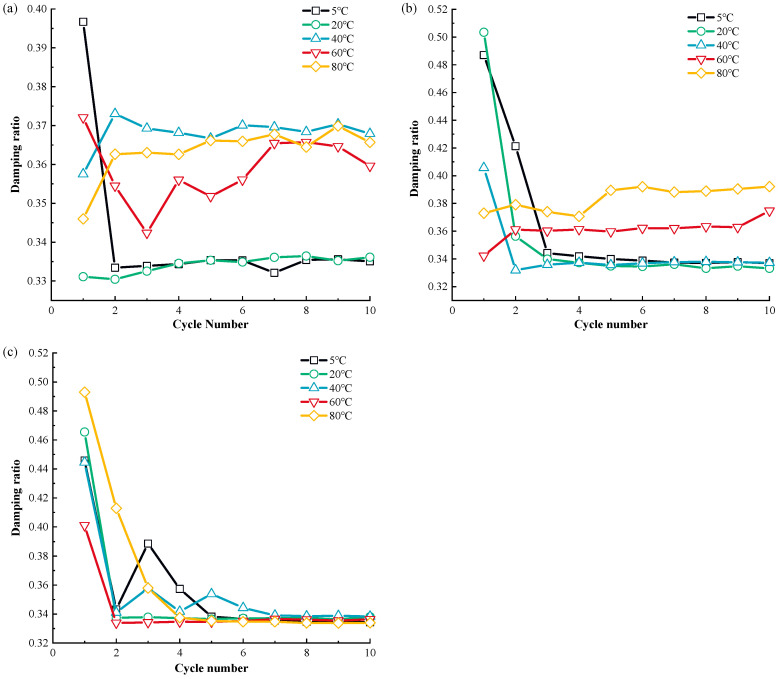
The relationship curve of MSW damping ratio and cycle number under different normal stress conditions: (**a**) 50 kPa; (**b**) 100 kPa; (**c**) 150 kPa.

The MSW damping ratio–cyclic shear frequency correlation curves are presented in Figure 6.

Based on Figure 6, as with the dynamic MSW shear stiffness, except for the damping ratio at 20 °C~40 °C, the variation in the damping ratio with varying rising temperatures is regular, and with the increase in temperature, the MSW damping ratio gradually goes up. For example, at 50 kPa, the damping ratios in the initial cycle are 0.30, 0.33, 0.34, and 0.36 at temperatures of 5 °C, 20 °C, 60 °C, and 80 °C, respectively. The reactivity of the MSW damping ratio to fluctuations in the 5 °C~20 °C range is lower than that in the 60 °C~80 °C range, in contrast to the dynamical MSW shear elasticity. For instance, at 100 kPa normal stress, in the initial shear cycle, the damping ratio increases by 2% from 0.49 to 0.50 when ascending from 5 °C to 20 °C, while the damping ratio rises by 8.82% from 0.34 to 0.37 when the temperature is elevated from 60 °C to 80 °C. It should be mentioned that the susceptibility of the MSW damping ratio to fluctuations in temperature decreases as the normal pressure rises. Taking the change in the MSW damping ratio at temperature ranges from 60 °C to 80 °C as an example, the damping ratio increases by 15.87% from 0.315 to 0.365, by 7.69% from 0.364 to 0.392, and by 5.99% from 0.334 to 0.336. Regarding the change law of the damping ratio during cyclic shear loading, it is found that, with a rise in normal stress, the difference between the damping ratio for different cyclic shear numbers rises. As an example, there is little variation in the damping ratio across various cyclic shear values at 50 kPa normal pressure. Comparatively, the damping ratio during the initial number is significantly greater than that in the subsequent cycle numbers at 150 kPa normal pressure.

### 3.3. The Temperature-Dependent MSW Mechanical Response in Post—Cyclic Monotonic Shear Loading

**Figure 7:**
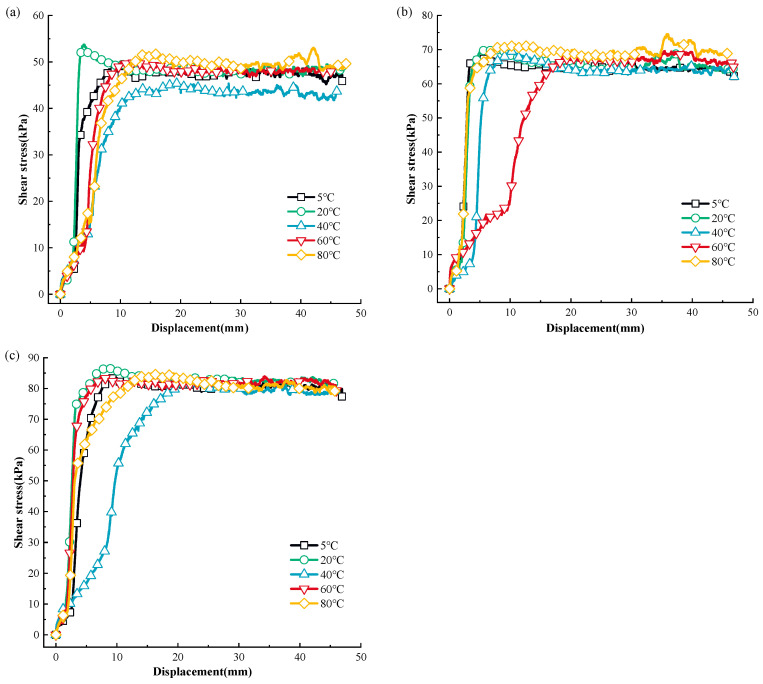
MSW shear stress–displacement curves under post-cyclic monotonic shear loading: (**a**) 50 kPa; (**b**) 100 kPa; (**c**) 150 kPa.

The correlation curves between the shear displacement and shear stress of MSW under post-cyclic monotonic shear loading at various temperatures are depicted in Figure 4, and the post-cyclic MSW peak shear strength–temperature relationship is shown in Figure 7.

**Figure 8:**
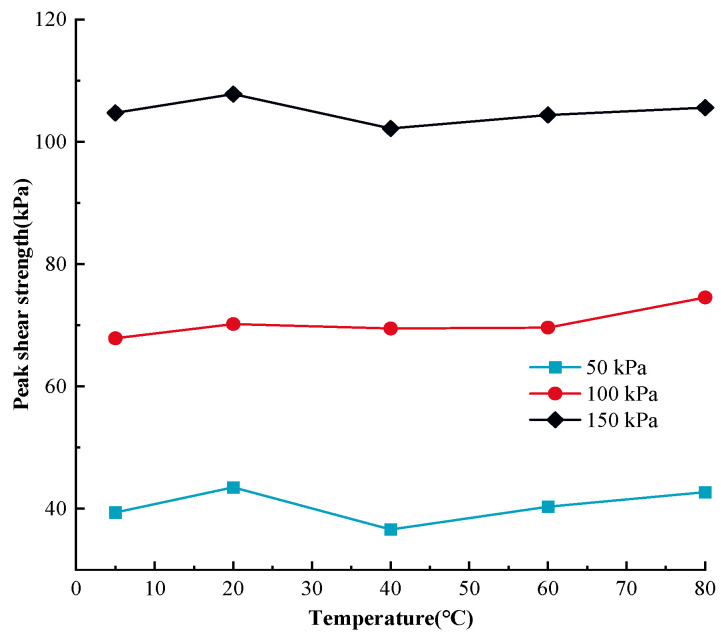
The correlation curves between temperature and MSW post-cyclical peak shear strength.

Based on Figure 7 and Figure 8, the post-cyclic MSW mechanical response presents significant temperature-dependency. As with the pre-cyclic MSW static mechanical response, at different normal pressures, the post-cyclic MSW peak shear strength rises at the temperature ranges of 5 °C~20 °C and 40 °C~80 °C, and the maximum shear strength decreases in the temperature range of 20 °C~40 °C. For instance, at 50 kPa normal pressure, the post-cyclic peak shear strength rises from 39.37 kPa to 43.47 kPa and from 36.58 kPa to 42.68 kPa in the temperature ranges of 5 °C~20 °C and 40 °C~80 °C, respectively, and the increases are 10.41% and 16.68%, respectively. The peak shear strength is reduced from 43.47 kPa to 36.58 kPa with a decrease of 18.84% in the temperature range of 20 °C~40 °C. Like the pre-cyclic MSW static mechanical response, the temperature-dependent post-cyclic shear strength reaches its maximum and minimum values at the temperatures of 20 °C and 40 °C, respectively. The post-cyclic peak shear strength is more dependent on the temperature range of 20 °C~40 °C than it is on other temperature ranges, in line with the pre-cyclic MSW mechanical parameters. For instance, at 150 kPa, the post-cyclic shear strength rises by 2.94% from 104.71 kPa to 107.79 kPa and by 3.33% from 102.17 kPa to 105.57 kPa when the temperature ascends from 5 °C to 20 °C and from 40 °C to 60 °C, while the shear strength decreases by 5.21% from 107.79 kPa to 102.17 kPa. It can be found that the difference in the peak shear strength at different temperatures decreases as the normal stress increases. This regularity is in agreement with the studies of Baker [96] and Vieira et al. [97].

### 3.4. The Comparison between Temperature-Dependent Static, Dynamic and Post-Cyclic Shear Mechanical Response of MSW

**Figure 9:**
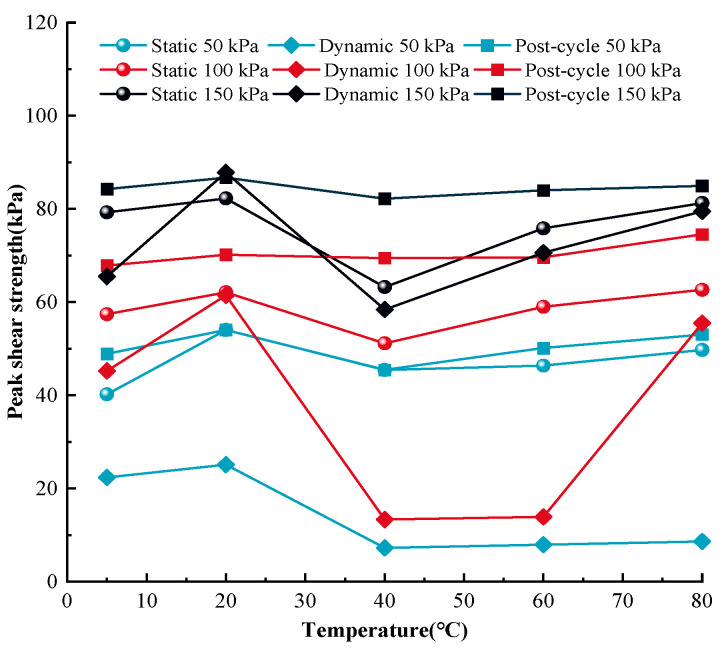
The MSW peak shear strength–temperature relationship curves for different stress statuses.

The peak shear strength of MSW under various stress statuses and temperatures are illustrated in Figure 9 in order to quantitatively compare the static, dynamic, and post-cyclic mechanical responses of MSW under monotonic, cyclic, and post-cyclic monotonic shear loading.

Based on Figure 9, in general, for the same temperature and normal stress, the post-cyclic MSW peak shear intensity is the highest, being followed by the pre-cyclic peak shear intensity, and the dynamic MSW peak shear intensity is the lowest. Additionally, the difference between the static and dynamic peak shear intensities rises when the normal stress decreases. For example, at a temperature of 60 °C, at normal stress of 50 kPa, the post-cyclic, pre-cyclic, and dynamic MSW peak shear strengths are 50.13 kPa, 46.36 kPa, and 7.96 kPa, respectively, and at normal stress of 150 kPa, the post-cyclic, pre-cyclic, and dynamic peak shear strengths are 83.98 kPa, 75.83 kPa, and 70.61 kPa, respectively. Regarding the variation rules of the MSW peak shear intensity, both of the post- and pre-cyclic peak shear intensities rise at temperatures ranging from 5 °C to 20 °C and from 40 °C to 80 °C, while the dynamic MSW peak shear intensity decreases at temperatures ranging from 5 °C to 20 °C and from 40 °C to 80 °C. The similarity of the static and dynamic mechanical responses is that the change law of the MSW peak shear intensity in the temperature range from 20 °C to 40 °C is different from that for other temperatures. Additionally, the sensitivity of the dynamic MSW shear strength to temperature variation is the largest, being followed by the post-cyclic peak shear intensity, and the pre-cyclic peak shear intensity is the lowest. For instance, under 100 kPa normal stress, the pre- and post-cyclic MSW peak strengths rise by 7.07% from 57.42 kPa to 61.48 kPa and by 3.42% from 67.86 kPa to 70.18 kPa, respectively, when the temperature rises from 5 °C to 20 °C; as well, they increase by 6.19% from 58.99 kPa to 62.64 kPa and by 7.09% from 69.60 kPa to 74.54 kPa, respectively, when the temperature ascends from 60 °C to 80 °C. The dynamic peak shear intensity decreases by 35.90% from 45.24 kPa to 61.48 kPa and by 298.63% from 13.92 kPa to 55.49 kPa when the temperature goes up from 5 °C to 20 °C and from 60 °C to 80 °C, respectively. Therefore, the dynamic peak shear strength decreases at temperatures increasing from 5 °C to 20 °C and from 60 °C to 80 °C, and the magnitude of the decline in shear strength ascends with increasing temperature. Similar results were obtained in a study by Shi et al. [98]. It also should be noted that the temperature-dependent pre-cyclic, post-cyclic, and dynamic peak shear strengths reach their maximum and minimum values at the temperatures of 20 °C and 40 °C, respectively. Taking the MSW peak shear strength under 100 kPa as an example, the post-cyclic, pre-cyclic, and dynamic peak shear strengths reach maximum values of 70.18 kPa, 61.48 kPa, and 60.19 kPa, respectively, at a temperature of 20 °C and reach minimum values of 69.45 kPa, 51.15 kPa, and 13.33 kPa, respectively, at a temperature of 40 °C.

There are multiple sources of potential error in the use of MSW for large-scale temperature-controlled direct shear tests, cyclic shear tests, and post-cyclic direct shear tests, which may affect the accuracy and reliability of the test results. Firstly, the collection method, location, and number of waste samples may affect the tests’ homogeneity and representativeness. Therefore, this study strictly controls the consistency of variables such as the waste composition, density, and water content at the Shanghai Laogang Landfill. The accuracy of the shear test equipment directly affects the accuracy of the measurement results. For example, the clearance of the shear box, the stiffness of the loading device, the sensitivity of the displacement transducer, and the precision of controlling the temperature may affect the test results. Therefore, in this study, high-precision and high-stability test instruments were selected, with regular calibration and maintenance. At the same time, the test conditions were strictly controlled to ensure the stability and accuracy of parameters such as the temperature, shear rate, and displacement. It is well worth noting that MSW may continue to degrade during the process of the test, which would result in changes in its physical and chemical properties. This could deviate the test results from the actual situation. Considering that there are very few studies taking this factor into account, future studies should consider the effects of specific components in the waste that affect the test results and establish relevant mechanical parameters to correct the experimental results. For example, Saberian et al. [99] proposed a reasonably feasible model for estimating the shear strength parameters of waste containing rubber crumbs.

## 4. Discussion

In this research, it is indicated that, at temperature ranges from 5 °C to 20 °C and from 40 °C to 80 °C, increases in the pre and post-cyclic static peak shear strengths of MSW occur, while, during at the temperature range of 20 °C~40 °C, the peak shear intensity of MSW declines. This can be attributed to the interlocking and sliding effects between different components of MSW being the major reason to generate the static peak shear strength [100,101]. Rises in temperature have different influences on the interlocking and sliding effects. Due to the softening of MSW resulting from increasing temperature, the diverse constitutions of MSW can be interlocked more deeply under the effect of normal stress to enhance the interlocking effects, which play an increasing role on the MSW static peak shear strength [102,103]. In comparison, the sliding effects between different components of MSW are weakened due to the softening of MSW in elevated temperatures, which plays a reducing role on the MSW peak shear strength [104]. Also, for different temperature ranges, the major factor for the MSW peak shear strength is different [73,105]. When the sliding effects are the dominating factor, the rising temperature can result in an increase in the MSW peak shear intensity. In contrast, when the interlocking effects are the main factor, the elevated temperature can lead to a decline in the MSW peak shear intensity [106]. It is inferred that, at temperatures ranging from 20 °C to 40 °C, sliding interactions are the dominating factor between different components of MSW. Thus, when the temperature rises in a range from 20 °C to 40 °C, the reduction in sliding effect results in the decline in the static MSW peak shear intensity. Comparatively, it is inferred that, in temperature ranges from 5 °C to 20 °C and from 40 °C to 80 °C, interlocking interactions are the major factor between different constituents of MSW. Thus, when the temperature elevates in these temperature ranges, the enhancement of interlocking impacts leads to the ascent of the MSW static peak shear intensity. 

Unlike the variation guidelines of static MSW mechanical features, in the temperature range of 5 °C~80 °C, the dynamic peak shear intensity of MSW continually reduces. This may be explained by the fact that, due to dynamic shear loading, the strong interlocking effects between different components of MSW are difficult to form during cyclic shearing. Thus, for the dynamic MSW mechanical response, the sliding effects between the diverse constituents of MSW are the major factor in generating dynamic peak shear intensity in the temperature range of 5 °C~80 °C. As mentioned, the sliding effects between different component of MSW are weakened due to the softening of MSW in elevated temperature, which plays a reducing role on the peak shear strength. This results in continual reduction in the dynamic MSW peak shear strength at temperatures ranging of 5 °C~80 °C. 

When comparing the temperature-dependent post-cyclic and pre-cyclic static MSW mechanical response, it is found that the post-cyclic peak shear intensity is greater than the pre-cyclic value between 5 °C and 80 °C, and that temperature variation has a greater impact on the post-cyclic peak shear intensity than the pre-cyclic strength. This is explained by the growing MSW compaction degree under cyclic shear stress, which strengthens the various MSW components’ interlocking and sliding effects [107,108,109,110,111,112,113,114,115]. In terms of the MSW peak shear strength, it strengthens. Furthermore, temperature influences the post-cyclic peak shear intensity more than the pre-cyclic peak shear intensity because of the increased interlocking and sliding effects between various MSW elements following cyclic shear loading. 

Regarding the change law of dynamic MSW mechanical properties during cyclic shear loading, it is discovered that the fluctuating mechanical features during the first-cycle shear loading are considerably different from the mechanical characteristics during the subsequent cyclic period. This could be explained by the fact that there are weak interlocking impacts between the various MSW components in the initial state due to the relatively loose MSW sample. MSW tightens up following the initial cyclic shear loading, intensifying the interlocking effects [110,111]. This is the reason for the glaring discrepancy between the fluctuating mechanical response of the initial shear cycle and that of the following cycles. Conversely, it is difficult to compact the sand much more during the ensuing shear cycles, and the difference in the interlocking impacts is negligible [93]. As a result, during the ensuing shear cycles, the dynamic MSW mechanical response is comparable.

The results of this study found that the mechanical properties of MSW subjected to monotonic, post-cyclic monotonic, and cyclic stress satisfy the engineering requirements and can be widely used as a construction material. Mechanical properties and stability studies on MSW can provide a basis for the subsequent design of mixed materials (foam concrete, recycled aggregates, eco-bricks, etc.). The results of this study will provide guidance on engineering design for the use of MSW. The mechanical properties of MSW at different temperatures were investigated; thus, the results of this study can provide guidance for engineering design and the application of MSW in different areas.

A preliminary study on different MSW from different zones/period of time could change some parameters. MSW from diverse zones and time periods should be considered in future studies to investigate the feasibility and applicability of MSW as a building material. In addition, as the main engineering scale of this paper is to study the physical properties of the waste, the chemical or phase compositions of the waste residue were not investigated in this paper, and will be studied in the future. Furthermore, toxic MSW exists, and the potential disposal of the toxic components may be challenging. In the future, research could be conducted on how to eliminate the toxicity of MSW after it has been used in construction materials.

## 5. Conclusions

Using a sizable temperature-controlled dynamic and static shear instrument that the authors constructed, an assortment of temperature-controlled static, dynamic, and post-cyclic shear experiments were performed on MSW in 5 °C~80 °C conditions in this work. As far as the authors are aware, there are not many papers discussing the temperature-dependent mechanical reaction of MSW, much less studies that take into account the impact of various stress states (monotonic, post-cyclic, and cycle shearing). According to the temperature-controlled test results, the static, dynamic, and post-cyclic shear mechanical responses of MSW subjected to monotonic, post-cyclic monotonic, and cyclic stress were systematically analyzed. 

The research outcomes indicate that, at temperatures ranging from 5 °C to 20 °C and from 40 °C to 80 °C, the pre-cyclic and post-cyclic static MSW peak shear strengths rise, while, at 20 °C~40 °C, a decrease in the peak shear intensity occurs. However, the dynamic MSW peak shear intensity, damping ratio, and shear rigidity continually increase at temperatures ranging from 5 °C to 80 °C. Also, the MSW mechanical response, both before and after cyclic static and dynamic testing, is more susceptible to fluctuations in temperature in the 5 °C~20 °C range than in the 40 °C~80 °C range. Additionally, in the 5 °C~80 °C range, the static MSW peak shear intensity is the highest, being followed by the post-cyclic peak shear intensity, while the dynamic peak shear intensity is the lowest. However, the sensitivity of the dynamic MSW shear strength to temperature variation is the largest, being followed by the post-cyclic peak shear intensity, and the static peak shear intensity is the lowest.

The mechanical properties of MSW at different temperatures were investigated. The results showed that MSW can be widely used as building material at 20 °C~80 °C. For the design of engineering projects within the temperature ranges that were given by this research, the results of this study will provide guidance for engineering designs and applications that use MSW. The effects on the mechanical properties of MSW in other temperature ranges will be studied in-depth in the future, and provide guidance on the suitability of different environments when using MSW as construction material.

## Figures and Tables

**Table 1:** Experimental scheme.

Experiment Type	Normal Stress (kPa)	Shear Rate (mm/min)	Shear Amplitude (mm)	Temperature (°C)
Monotonic direct shear test	50 100 150	1.0	55.0	5 20 40 60 80
Cyclic shear test	50 100 150	1.0	3.0	5 20 40 60 80
Post-cyclic direct shear test	50 100 150	1.0	55.0	5 20 40 60 80

## Data Availability

In this paper, all data and models used during the research appeared in the submitted manuscript.
